# Synergistic application of melatonin and silicon oxide nanoparticles modulates reactive oxygen species generation and the antioxidant defense system: a strategy for cadmium tolerance in rice

**DOI:** 10.3389/fpls.2024.1484600

**Published:** 2024-10-15

**Authors:** Mohammad Faisal, Mohammad Faizan, Sipan Soysal, Abdulrahman A. Alatar

**Affiliations:** ^1^ Department of Botany & Microbiology, College of Science, King Saud University, Riyadh, Saudi Arabia; ^2^ Botany Section, School of Sciences, Maulana Azad National Urdu University, Hyderabad, India; ^3^ Department of Field Crops, Faculty of Agriculture, Siirt University, Siirt, Türkiye

**Keywords:** abiotic stress, oxidative damage, protein content, superoxide dismutase, total free amino acids, total soluble sugar

## Abstract

Unfavorable environmental conditions pose a major barrier to sustainable agriculture. Among the various innovative strategies developed to protect plants from abiotic stress, the use of phytohormones and nanoparticles as “stress mitigators” has emerged as one of the most important and promising approaches. The objective of this study was to observe the protective role of melatonin (Mel) and silicon oxide nanoparticles (SiO-NPs) in rice (*Oryza sativa* L.) seedlings under cadmium (Cd) stress. Rice seedlings have reduced growth and phytochemical attributes when grown in Cd-contaminated (0.8 mM) pots. Seedlings under Cd stress had 38% less shoot length (SL), 53% total soluble sugar (TSS) and 57% protein content. However, superoxide dismutase (SOD), hydrogen peroxide (H_2_O_2_) and malondialdehyde (MDA) increased by 51%, 37% and 34%, respectively, under Cd stress. Beside this, activities such as peroxidase (POX) also elevated in the plants subjected with Cd-stress. In contrast, Mel (100 µm) as foliar spray and SiO-NPs (100 mg/L) as root dipping reduced oxidative stress in rice seedlings under Cd stress by reducing reactive oxygen species (ROS) generation. Furthermore, the application of Mel and/or SiO-NPs significantly increased the activity of antioxidative enzymes that scavenge ROS. The combined application of SiO-NPs and Mel increased growth, gas exchange and photosynthetic attributes, chlorophyll value, and protein content. It causes alleviation in the activity of SOD, CAT and POX by 73%, 62% and 65%, respectively. Overall, this study findings show that Mel and/or SiO-NPs can potentially protect the rice crop against oxidative damage under Cd stress.

## 1 Introduction

Heavy metals (HMs) are not necessary for humans, animals, or plants; they can have detrimental effects if exposure to them exceeds certain minimum threshold values (Jiang et al., 2023). Heavy metals are non-biodegradable; they build up in the soil and significantly inhibit plant growth through cytotoxicity and genotoxicity. Certain HMs restricts the growth of plants by disrupting the activity of hydrolytic enzymes and α-amylase proteases (Seneviratne et al., 2019). Additionally, HMs accumulate more in newly formed tissues, hindering the uptake of carbohydrates and the transfer of sugars to developing cells (Devi et al., 2007; Sfaxi-Bousbih et al., 2010). Over 10 million sites are classified as polluted globally, with over half of them affected by metalloids like arsenic or HMs like Cd, lead, and aluminum.

Cadmium is one of the most notable elements in the HMs spectrum due to its widespread abundance and classification as a highly hazardous non-essential element (Li et al., 2022). It enters the environment through natural processes as well as increased human activities like mining, industrial processes, disposing of waste containing metals like batteries, sludge disposal, over fertilizing, and using products related to Cd. Prolonged exposure to Cd interferes with photosynthetic and enzymatic processes, damaging membranes, and reduces plant growth and seed germination (Kumar et al., 2024). There are serious concerns to human health and the food chain from this increased release of Cd into the environment. Due to insufficient regulatory mechanisms, plants are seeing an alarming increase in Cd bioaccumulation, which puts higher trophic organisms including humans at further risk (Yuanyuan et al., 2022; Jianchao et al., 2023; Yasin et al., 2024). Effective and creative remediation techniques are urgently needed to address the toxicity of Cd for human health and global food safety. The current study provides an environmentally friendly way to manage Cd toxicity by regulating protective photosynthetic attributes and enhancing antioxidant machinery.

Melatonin is a molecule that functions in plants as a plant hormone, controlling their growth and development and assisting them in fending off abiotic stress (Meenu et al., 2024). The hormone Mel is a byproduct of the tryptophan metabolic process. Plant-derived Mel is referred to as Mel (N-acetyl-5-methoxytryptamine). Almost all plant species analyzed since Mel discovery in 1995 have been found to contain it. However, Mel levels vary widely throughout plant species. Melatonin is produced by plant cellular organelles called mitochondria and chloroplasts. The cytoplasm and endoplasmic reticulum are known to have enzymes that synthesize Mel, indicating that these organelles are involved in the generation of Mel (Meenu et al., 2024). Melatonin has been proven to be continuously produced by plant tissues (Wang et al., 2014). It is mentioned that Mel is a pleiotropic hormone with a wide range of beneficial effects that generally improve physiological processes like water content, seed and fruit yield, osmoregulation, and control of different metabolic pathways like the sulfur, phosphorus, carbohydrates, and lipids cycles. In addition, stomatal conductance, pigment concentration, and photorespiration (components of photosynthesis) also influenced by Mel (Faizan et al., 2024a; Hussain et al., 2024). In connection with secondary metabolism, Mel promotes the synthesis of various terpenoids, flavonoids, anthocyanins, carotenoids, and simple phenols. Melatonin should therefore play a role in regulating the growth and development of plants. It has been found to be effective in plants for increasing chlorophyll’s photosynthetic activity while maintaining redox homeostasis, in addition to aiding in the maintenance of chlorophyll. Melatonin provides resistance to HMs during growth by controlling multiple physiological processes, such as an increased glutathione pool, increased amounts of Mel inside the plant, the restoration of redox equilibrium, and increased quantities of reducing and soluble sugars within germinating seeds (Arnao et al., 2021). According to Weeda et al. (2014), Mel increases the transcript levels of several defense-related components, including as transcription factors, kinases, and stress receptors. Melatonin has been shown, for instance, to encourage the formation of coleoptiles in four monocot species: canary grass, wheat, barley, and oat (Hernandez-Ruiz et al., 2005). Additionally, Mel stimulates adventitious root regeneration in sweet cherry shoot tip explants (Sarropoulou et al., 2012) and root growth in *Brassica juncea* (Chen et al., 2009). Tan et al. (2012) reported that Mel-treated corn plants yielded higher yields compared to untreated ones.

Nanotechnology offers novel methods for fostering tolerance to abiotic stress and boosting crop output, it is a creative way to improve the agriculture sector (Elemike et al., 2019; Alabdallah et al., 2021a; Faizan et al., 2024a). Molecules with sizes ranging from 1 to 100 nm are known as nanoparticles (NPs). Their high surface energy and surface-to-volume ratio are necessary for biological activity, and they have a range of physicochemical characteristics, including enhanced reactivity (Mittal et al., 2020; Bhat et al., 2023). When used as herbicides, nanopesticides, and nanofertilizers, among other things, it can effectively release its material in the right amounts to target plant cellular organelles, which can aid in the promotion of plant morphogenesis (Alabdallah et al., 2021b). Different NPs have been identified for their potential in alleviating HM-induced toxicity in different plants through improved nutrient uptake, enhanced photosynthesis, cellular metabolite regulation, and enzymatic and non-enzymatic antioxidant activation (Faizan et al., 2021; Bhat et al., 2022; Faizan et al., 2023; Liu et al., 2023). Silicon as a nanomaterial has drawn a lot of interest in the field of agrochemicals and may be a more effective way to relieve various abiotic stressors than bulk material (Rastogi et al., 2019; Goswami et al., 2022; Faizan et al., 2024b). According to reports, Si NPs have the ability to protect *Cucumis sativus* plants from salinity stress in terms of their morphological characteristics and metabolic control (Avestan et al., 2019). Similarly, via controlling plant morpho-physiological and antioxidant characteristics as well as the synthesis of certain metabolites, the use of Si NPs has demonstrated excellent potential for the post drought recovery of *Hordeum vulgare* (Ghorbanpour et al., 2020). Recent studies have demonstrated that Si NPs can increase plant resistance to HM stress by altering the ultrastructure of cells, antioxidant enzymes, and ROS (Emamverdian et al., 2020). Si NPs can also reduce stress by modifying transduction processes and signaling pathways through interaction with ROS and associated gene activity (Mukarram et al., 2022).

Rice (*Oryza sativa*) is one of the main staple food, widely grown throughout the globe, and almost half of the world’s population is dependent upon rice (Sen et al., 2020; Hasnain et al., 2023), and more than a hundred nations grow it, with Asia producing 90% of the world’s rice (Fukagawa and Ziska, 2019). Climate change affects the world’s food supply, consumer accessibility, and food quality, which leads to unsustainable agriculture (Adil et al., 2020). The efficient absorption of Cd by rice plants has made Cd a significant concern in rice production systems (Ismael et al., 2019). The morphological and physiological characteristics of rice plants are hampered by the transfer of Cd, which eventually lowers grain output. These characteristics include plant height, root length, leaf size, and biomass (Rahman et al., 2023; Shaghaleh et al., 2024). In addition to lowering the nutritional value of rice, its accumulation in rice grains puts consumers at danger of Cd poisoning (Rahman et al., 2023). Additionally, by interfering with non-specific transporters like Zn^2+^ and Ca^2+^ channels, Cd upsets ionic homeostasis (Adil et al., 2020). In order to improve crops while preserving environmental balance, modern agricultural production methods aim to use sustainable approaches. Using NPs (Rizwan et al., 2019) and phytohormones (Munir et al., 2024) is one of the finest method of reducing the toxicity of Cd in plants.

It is yet unclear how Mel synergistically with SiO-NPs treatment affects rice growth, photosynthesis and antioxidant enzymes under Cd stress. Thus, the primary goal of the current study is to better understand how Mel and SiO-NPs interact together to lessen the effects of Cd stress and encourage rice growth, photosynthesis, and antioxidant enzyme activity along with reducing oxidative stress. Furthermore, it is crucial to investigate in future research projects how Mel and SiO-NPs cooperate to regulate the different physiological pathways during rice growth under Cd stress. This study offers novel perspectives in an environmentally responsible way while addressing the urgent worldwide issue of food safety and security.

## 2 Material and methods

The rice (*Oryza sativa* L.) seeds were purchased from local market of Hyderabad-500032, India. The seeds were treated with 10% (w/v) sodium hypochlorite (NaClO) solution for 15-20 min followed by 3-4 times washing with double distilled water (DDW). To ensure resilience each treatment was repeated three times. In order to facilitate seed germination, the incubator (Blue Pard MGC-HP) was kept at a constant temperature of 25°C. Three-leaf stage seedlings were moved into the maintained pots.

### 2.1 Treatments

**Figure 1:**
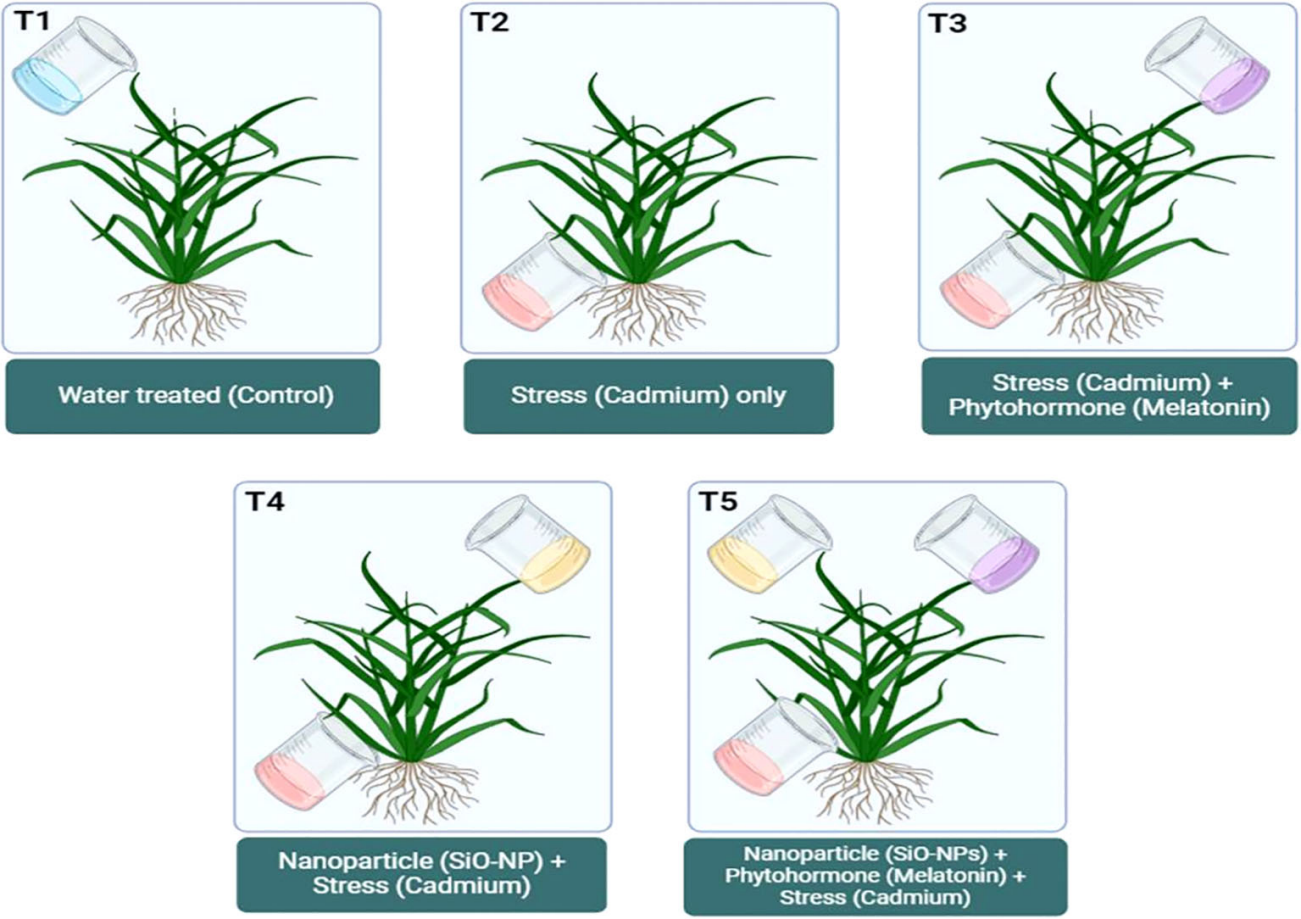
Diagrammatic representation of experimental setup.

During experiment, cadmium chloride (CdCl_2_) was applied through the soil for the purpose of Cd toxification. On the other hand, Mel was applied topically, and SiO-NPs were subjected by the root. At the time of seedlings transplanting, Cd stress (0.8 mM) was administered to the soil as a treatment (Cd concentration was selected on the basis of my previous study; Faizan et al., 2021). Chemically synthesized SiO-NPs was procured from Adnano Technologies Private Limited, KIADB Machenahalli Industrial Area, Shimoga-577000, Karnataka, India, and required concentration (100 mg/L) of SiO-NPs was prepared by diluting the stock solution. The roots of the seedlings were immersed in SiO-NPs (100 mg/L) for 15 minutes at the time of transplantation in to the pots; however, plants at 35 days old were supplied with Mel (100 µm) treatment to their foliage (continuously five days) at evening, while control plants were applied with DDW. Therefore, there were five treatments in total in this study: (1) Control: treated only DDW; (2) Cd: Soil treated with 0.8 mM of CdCl_2_; (3) Cd + Mel: Soil treated with 0.8 mM of CdCl_2_ and leaves treated with 2 mL of 100 µm Mel; (4) Cd + SiO-NPs: Soil treated with 0.8 mM of CdCl_2_ and root dipped in 10 mL of 100 mg/L SiO-NPs; (5) Cd + Mel + SiO-NPs: Soil treated with 0.8 mM of CdCl_2_, leaves treated with 2 mL of 100 µm Mel and root dipped in 10 mL of 100 mg/L SiO-NPs (**Figure 1**). The experiment involved rice plants cultivated in Cd-contaminated soil, with three replicates in a complete randomized design. At the 40-day stage, plants were harvested, and arbitrary samples were collected to evaluate the different growth and physio-biochemical features.

### 2.2 Characterization of SiO-NPs

**Figure 2:**
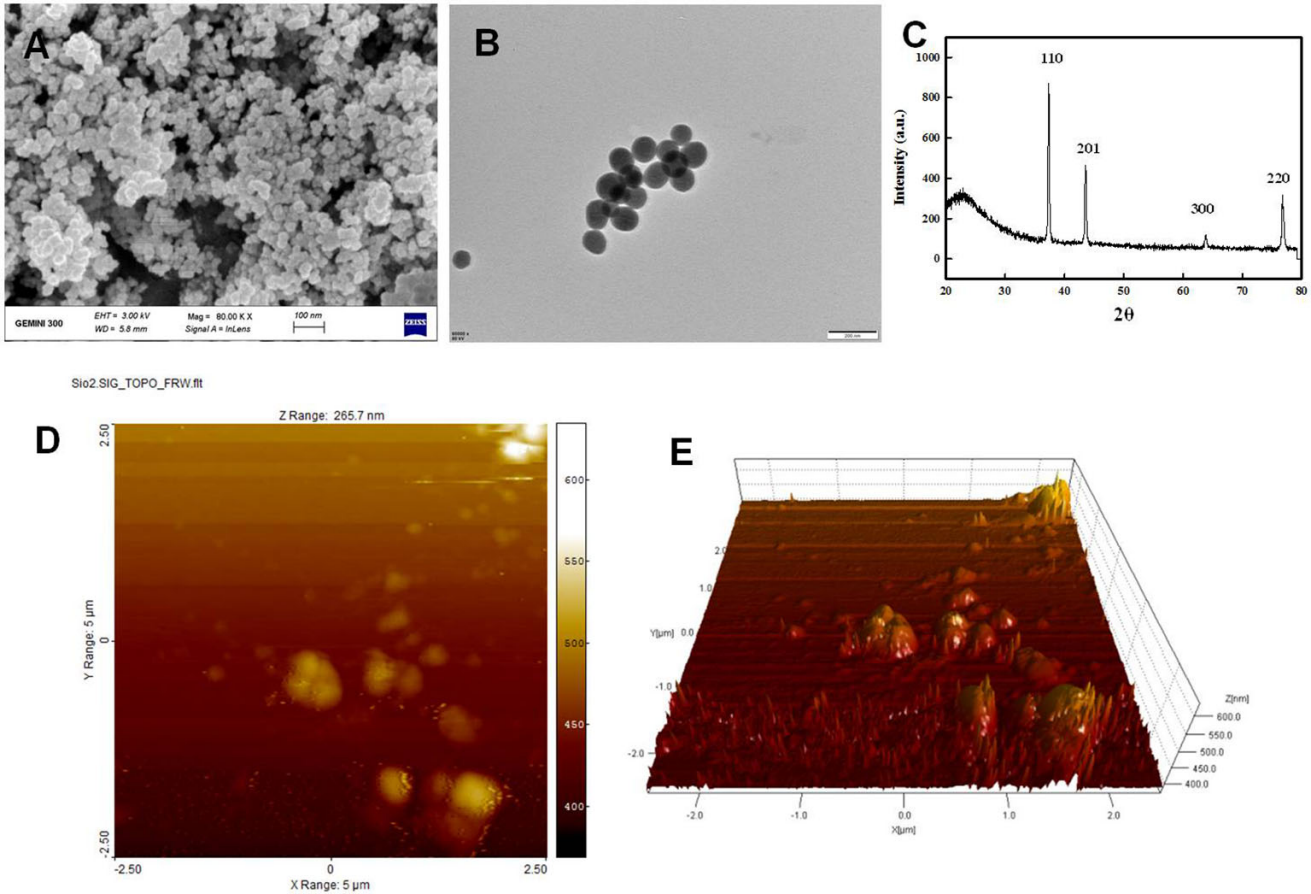
Characterization of SiO-NPs, **(A)** TEM: transmission electron microscopy, **(B)** SEM: scanning electron microscopy, **(C)** XRD: The X-ray diffraction pattern, **(D, E)** AFM: Atomic force microscope showing the 2-D and 3-D topography.

SiO-NPs were characterized morphologically and structurally using transmission electron microscope (TEM) and scanning electron microscope (SEM) investigations, as illustrated in **Figure 2**. TEM (JEM-1230, JEOL, Akishima, Japan) and SEM (TM-1000, Hitachi, Japan) were used to examine the surface morphology and particle size of SiO-NPs; however, the TEM image revealed that the SiO-NPs have a spheroidal form with an average size of 30 nm and length of 0.2 µm (**Figure 2A**). Additionally, the homogeneous distribution of the particles was seen in the SEM image (**Figure 2B**). While a 200-mesh carbon-coated copper grid was utilized for TEM investigation, the NPs were attached on an aluminum stub for SEM analysis using carbon double-sided glue tabs and encased with thin conductive palladium layer. Additionally, SiO-NPs crystallite structure was examined using x-ray diffraction (XRD) analysis (**Figure 2C**) in accordance with Nabila and Kannabiran (2018), with data acquired using 2θ ranging from 20 to 80°. The diffraction peaks at (110), (201), (300) and (230) revealed in the XRD pattern of the pure SiO-NPs planes clearly show their crystalline nature are shown. Characterizations of SiO-NPs were further done by analyzing the surface topography of powdered SiO-NPs using atomic force microscope (AFM) (Veeco Instruments, USA) in noncontact tapping mode. The topographical images were obtained in tapping mode with a resonance frequency of 218 kHz (**Figure 2D, E**).

### 2.3 Measurement index

#### 2.3.1 Phenotypic characteristics

Each plant’s shoot & root length, and their fresh & dry weight were measured with a ruler. An electronic balance was used to weigh the aboveground and belowground portions. From every triplicate, a single plant was chosen, baked for 30 minutes at 105°C, and then dried at 80°C until it reached a consistent weight.

#### 2.3.2 Photosynthetic rate and chlorophyll content

Various photosynthetic parameters were measured using a portable photosystem (model LI-COR 6400, LI-COR, Lincoln, NE, USA). We used this equipment to measure the net photosynthetic rate (P_N_), stomatal conductance (gs), intercellular CO_2_ concentration (Ci) and transpiration rate (E) values in the plant’s completely grown leaves. The variables that were maintained were the air temperature, relative humidity, CO_2_ concentration, and photosynthetic photon flux density (PPFD), which was 25 ~C, 85%, 600 µmol mol^-1^, and 800 µmol mol^-2^ s^-1^, respectively. Using a Minolta chlorophyll meter (SPAD-502; Konica Minolta Sensing Inc., Tokyo, Japan), the amount of chlorophyll was measured as the SPAD value.

#### 2.3.3 Assessment of the activity of antioxidant enzymes

Fresh leaves were considered for the enzyme assay. With a small adjustment, all of the required chemical and enzyme-extracting solutions were made using the techniques provided by Arya et al. (2022). 1 g of fresh leaf samples were first homogenized in enzyme extraction buffer. The supernatant was then collected and utilized for the measurement of SOD, CAT and POX activities. The preparation of a reaction mixture for CAT analysis included phosphate buffer, hydrogen peroxide (H_2_O_2_, 0.1 M), and enzyme extract (0.1 ml). Following a one-minute addition of sulfur dioxide (H_2_SO_4_), the mixture was titrated with a potassium permanganates solution (Chance and Maehly, 1956). In order to determine POX activity, 0.1 ml of the enzyme extracts were combined with a 1% solution of phosphate buffer, pyrogallol, and H_2_O_2_, and the absorbance at 420 nm was measured using a spectrophotometer (Chance and Maehly, 1956). The following mixture was made for the measurement of SOD activity: 50 mM of phosphate buffer, 20 μM of riboflavin, 75 mM of NBT, 13 mM of methionine, and 0.1 mM of ethylene diamine tetra acetic acid. In order to measure the absorbance at 560 nm, this combination was subjected to fluorescent light for irradiation (Beauchamp and Fridovich, 1971).

#### 2.3.4 Proline content

A method developed by Bates et al. (1973) was utilized to determine the proline content of freshly produced leaves. An equivalent volume of glacial acetic acid and ninhydrin solutions were applied to leaves that had been extracted in sulfosalicylic acid. After heating the sample to 100°C, 5 mL of toluene was added. Using a spectrophotometer, the absorbance of the aspirated layer was measured at 528 nm.

#### 2.3.5 Analysis of MDA and H_2_O_2_

To determine the quantity of MDA, 0.5 g leaf samples were homogenized in 10% trichloroacetic acid (TCA), centrifuged at 12,000 g for 10 min, combined with an equivalent amount of 0.67% 2-thiobarbituric acid (TBA), heated for 30 minutes at 100°C, and then cooled in an ice bath. Attenuation coefficient of 155 mM^−1^ cm^−1^ was used to evaluate the levels of MDA at OD 532 and 600 nm (Khan et al., 2019). The technique outlined by Velikova et al. (2000) was used to quantify the concentration of H_2_O_2_. The H_2_O_2_ content of the samples was determined using a spectrophotometer (Beckman 640D, USA), and the absorbance was measured at 390 nm.

#### 2.3.6 Protein estimation

To determine the amount of protein, Bradford’s (1976) method was used. To estimate the amount, 1 gram of fresh leaves was homogenized in a buffer containing 40 milligrams of tris-HCl (pH 7.5), 0.07% β-mercaptoethanol, 2% polyvinylpyrrolidone, 0.5% Triton X-100, 1 milligram of phenyl methane sulfonyl fluoride (PMSF), and 1 milligram of EDTA. The mixture was then centrifuged at 20,000 g for ten minutes. After gathering the supernatant, Bradford reagent was applied to facilitate color development. Protein content was reported as mg g^−1^ (FW) after absorbance was measured in a spectrophotometer.

#### 2.3.7 Determination of total soluble sugar and total free amino acids

Fresh leaves of rice plants that had been treated with Mel and SiO-NPs under Cd stress were homogenized in 1% acidic methanol (5 mL) and centrifuged for 10 min at 14,000 rpm. Then, a new tube was filled with the supernatant and 3 mL of anthrone reagent. The reaction mixture was cooled to room temperature following a 10-minute heating period at 95°C. Ultimately, Liu et al. (2018) method was used to assess the absorbance at 595 nm through a UV-visible spectrophotometer. Plant leaves homogenized in 1% acidic methanol (5 mL) and centrifuged for 10 min at 14,000 rpm. The supernatant was transferred to a new tube and mixed with 3 mL of anthrone reagent. The reaction mixture was brought to room temperature following a 10-minute heating period at 95°C. Finally, using a UV-visible spectrophotometer, the absorbance at 595 nm was detected (Crampton et al., 1957).

#### 2.3.8 The elemental analysis of Cd in the roots and shoots

To get the dry weight, the entire plant was crushed, pulverized, and weighed. Following a 12-hour nitration period using 0.05 g of sample and 20 mL of nitric acid: perchloric acid (3:1), the tetrafluoro crucible was put on an electric heating plate in a fume hood, approximately 170°C, to eliminate the acid. The solution was cooled, fixed at a 25 mL volume with 3% nitric acid, agitated thoroughly, and stored for later use when the reaction reached the size of a soybean. The Cd content was ultimately ascertained by the utilization of Perkin Elmer (PE) Optima 2100 DV inductively coupled plasma emission spectrometer.

#### 2.3.9 Scanning electron microscopy

Plant leaves’ stomatal apertures were examined using a scanning electron microscope (JEOL, JSM 6510). Fresh leaves were gathered and preserved for two hours using 0.1 M sodium cacodylate buffer (pH 7.3), 2.5% glutaraldehyde, and 2% paraformaldehyde. To go through the ethanol graded series (50, 70, 80, 90, and 100%), the leaves were moved to Petri plates. Samples were dried and then coated in gold–palladium using a JEOL JFC-1600 sputter coater.

#### 2.3.10 Statistical analysis

Analysis of variance (ANOVA) was conducted followed by Tukey’s test for *post hoc* analysis, using SPSS v18.0 for Windows (IBM Corporation, New York, NY, USA) at a *p* < 0.05 level.

## 3 Results

### 3.1 TEM, SEM, XRD and AFM micrograph

**Figures 2A, 2B** display TEM and SEM pictures of SiO-NPs. These pictures demonstrated the aggregate type of SiO-NPs, their distribution is random, and they have few extra pores on the specimen surface. Their form resembles cauliflower and they are highly related to one another. For plant use and catalysis, this form of morphology is considered ideal. The reasons are that these structures provide a larger surface area and are easily able to pass through plant barriers (membranes and cell walls) to target plant molecules (Motzkus et al., 2013). This increases the surface’s reaction and further leads to the sensor responding selectively at intrinsic standard level (Motzkus et al., 2013). The X-ray diffraction pattern of SiO-NPs is shown in **Figure 2C**. The peaks were indexed using Powder X software and were found corresponding with the structure of SiO (JCPDS card No. 46-1045).The estimated size corresponding to the most intense crystallographic plane (110) was determined to be 31.5 nm. **Figures 2D, E** showing the Atomic force microscope (AFM) image analysis of SiO-NPs showing the 3D topography of NPs. Average size of SiO_2_NP has been estimated to be 42 nm.

### 3.2 Exogenous application of Mel and SiO-NPs on phenotypic characteristics under Cd stress

**Figure 3:**
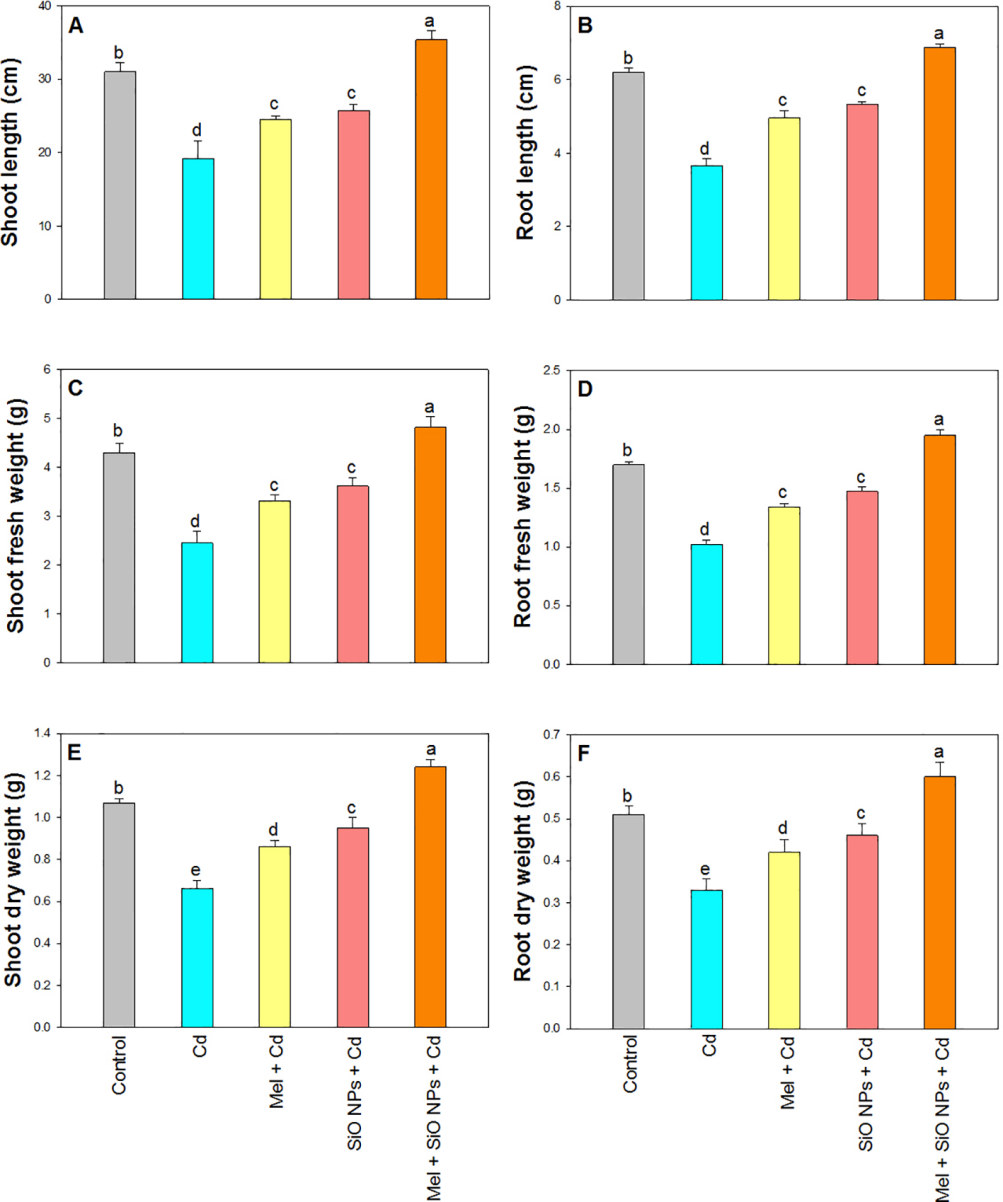
Exogenous application of SiO-NPs and Mel on 40-days old rice plants under Cd stress. **(A)** Shoot length; **(B)** Root length; **(C)** Shoot fresh weight **(D)** Root fresh weight; **(E)** Shoot dry weight, and **(F)** Root dry weight. The data is shown as the average of three sets ± Standard Error (S.E.). Different letters in graphs denote significant differences between control and treatment at P < 0.05 (Tukey's test).

Growth attributes of rice plant i.e. SL, RL, SFW, RFW, SDW, and RDW presented in **Figure 3**. A significant decrease in all the growth parameters was recorded under Cd stress at SL for 38%, RL for 41%, SFW for 43%, RFW for 40%, SDW for 38%, and RDW for 35% compared to their control plants. Root dipping of SiO-NPs and foliar application of Mel alone as well as in combination increased all these parameters under stress. The maximum value was recorded in the plants which received both SiO-NPs and Mel under Cd stress i.e. 14% for SL, 11% for RL, 12% SFW, 15% RFW, 16% RFW, and 19% for RDW over their control plants (**Figures 3A-F**).

### 3.3 Photosynthetic rate and chlorophyll content in the presence of Mel and SiO-NPs under Cd stress

**Figure 4:**
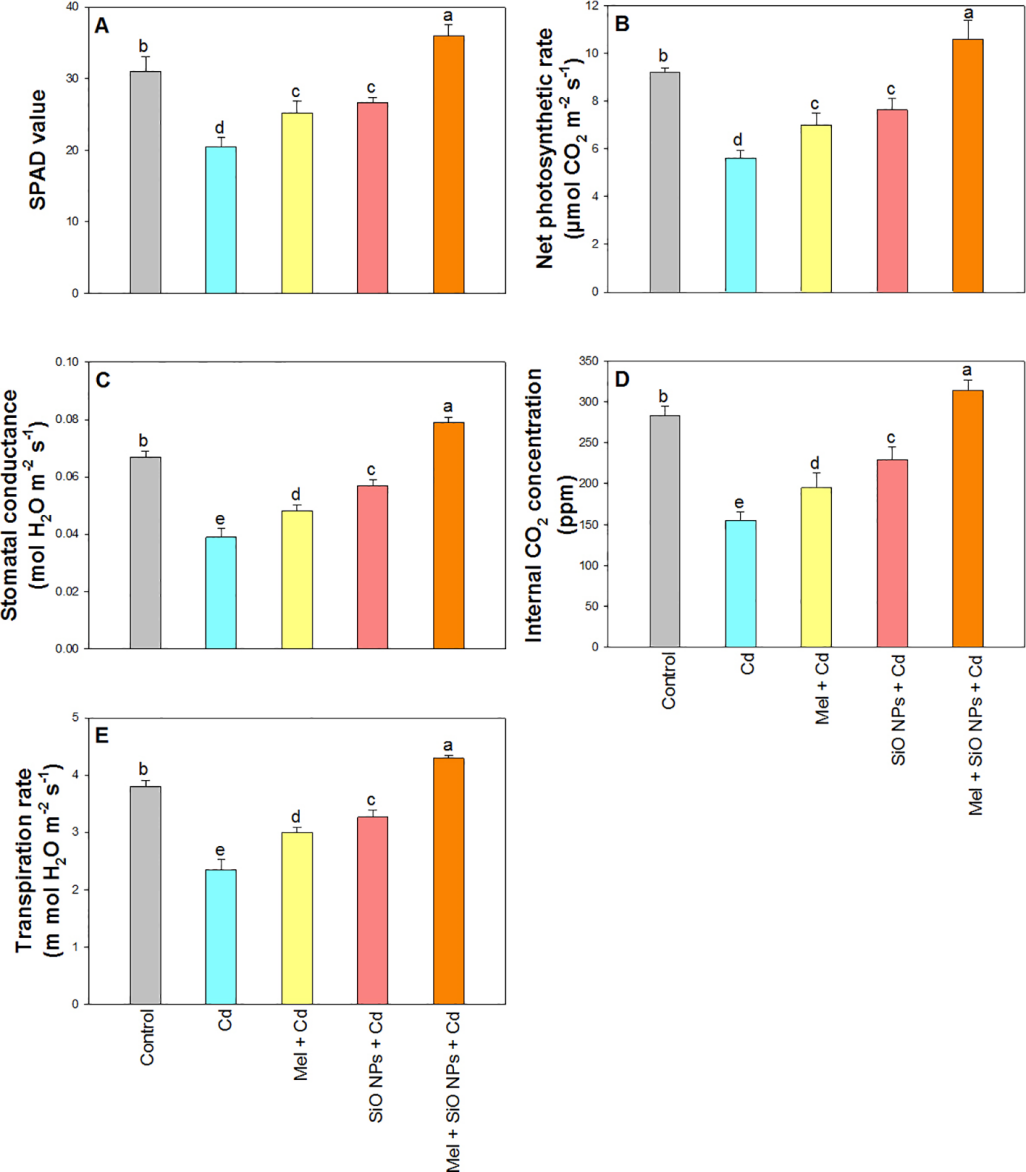
Exogenous application of SiO-NPs and Mel on 40-days old rice plants under Cd stress. **(A)** SPAD value; **(B)** Net photosynthetic rate; **(C)** Stomatal conductance **(D)** Internal CO_2_ concentration; **(E)** Transpiration rate. The data is shown as the average of three sets ± Standard Error (S.E.). Different letters in graphs denote significant differences between control and treatment at P < 0.05 (Tukey’s test).

The photosynthesis was summed up in **Figure 4** along with associated characteristics including P_N_, gs, E, Ci and chlorophyll content. In comparison to their respective controls, all photosynthetic qualities were considerably decreased in the presence of Cd at P_N_ for 39%, gs for 41%, Ci for 45%, and E for 38%. On the other hand, Mel applied exogenously and SiO-NPs as root dipping considerably decreased the toxicity of Cd in rice plants. Under Cd stress, the addition of SiO-NPs and Mel was found to maximum enhancements in photosynthetic characteristics at P_N_ for 15%, gs for 18%, Ci for 11%, E for 13%, and chlorophyll content for 16% compared to their controls (**Figures 4A-E**).

### 3.4 Melatonin and SiO-NPs affect the activity of antioxidant enzymes in the context of Cd toxicity

**Figure 5:**
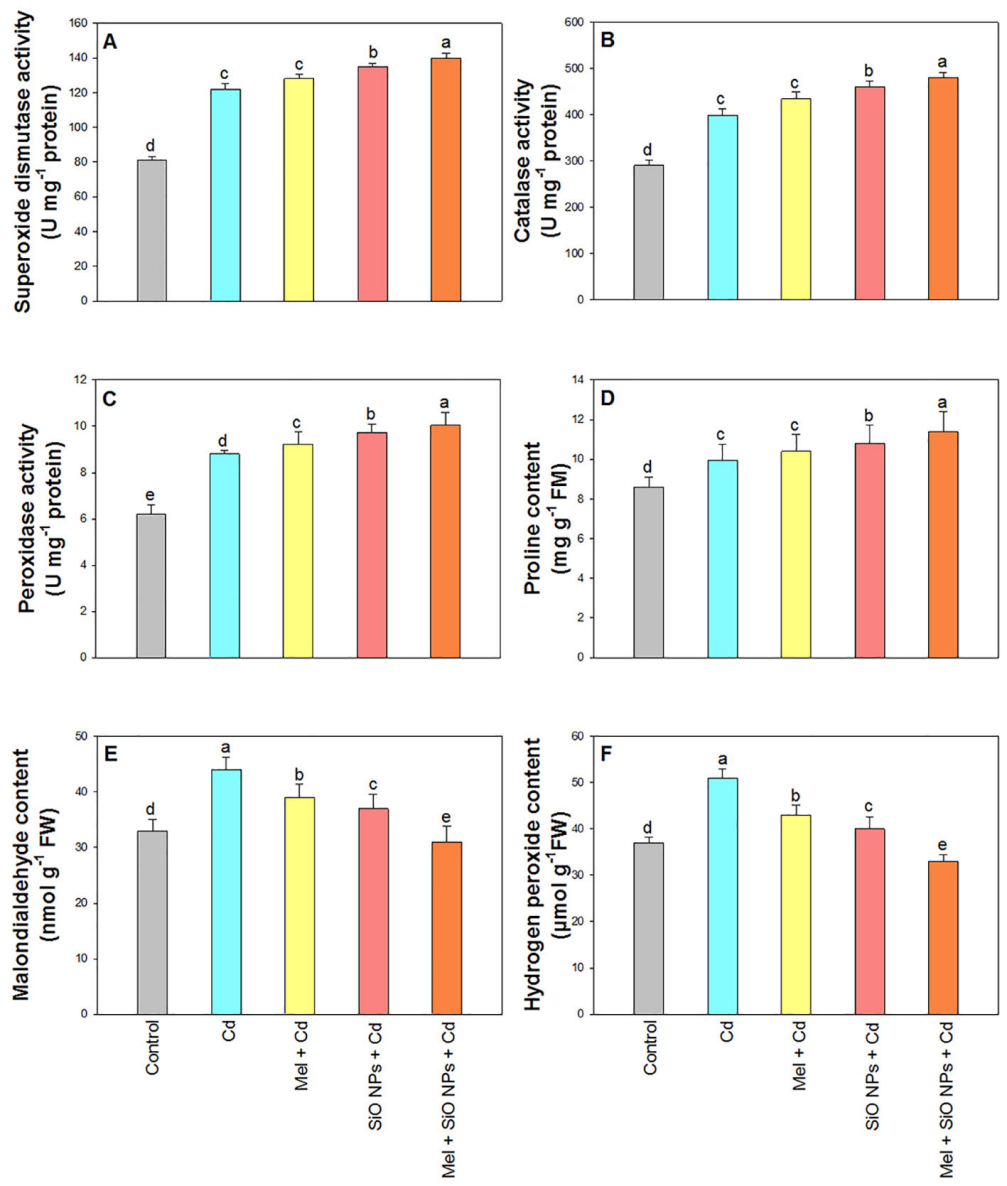
Exogenous application of SiO-NPs and Mel on 40-days old rice plants under Cd stress. **(A)** Superoxide dismutase activity; **(B)** Catalase activity; **(C)** Peroxidase activity **(D)** Proline content; **(E)** Malondialdehyde content, and **(F)** Hydrogen peroxidase content. The data is shown as the average of three sets ± Standard Error (S.E.). Different letters in graphs denote significant differences between control and treatment at P < 0.05 (Tukey’s test).

**Figure 5** presents findings about the activity of three important antioxidant enzymes (SOD, CAT, and POX) in rice leaves. When compared to control plants, Cd poisoning markedly raised the activity of SOD (51%), CAT (37%), and POX (42%). When compared to their respective Cd-only treatments, the application of SiO-NPs and Mel further increased the activity of these antioxidant enzymes. The plants that were exposed to Cd stress and that received Mel and SiO-NPs exhibited the highest activity of these enzymes, with increases of 73% (SOD), 65% (CAT), and 62% (POX) relative to the control group (**Figures 5A-C**).

### 3.5 Variations in proline content following Mel and SiO-NPs treatments under Cd stress

Proline content in rice leaves was increased (7%) in the presence of Cd (**Figure 5D**). This content gradually increased in the plants treated with Mel and SiO-NPs as compared to control plants. In contrast, the plants that got both Mel and SiO-NPs in addition to Cd stress had the highest proline concentration (16%).

### 3.6 Melatonin and SiO-NPs effects on MDA and H_2_O_2_ under conditions of Cd toxicity

Cd stress significantly increased the MDA and H_2_O_2_ content in leaves of rice compared to control (**Figures 5E, F**). Melatonin and SiO-NPs significantly decreased the MDA (19% and 13%) and H_2_O_2_ (17% and 9%) content as compared to control. Furthermore, the toxicity of MDA and H_2_O_2_ was totally neutralized by the combined application of Mel and SiO-NPs, which reduced their respective toxicity levels by 6% and 11%.

### 3.7 SiO-NPs and Mel effect in protein content under Cd stress

**Figure 6:**
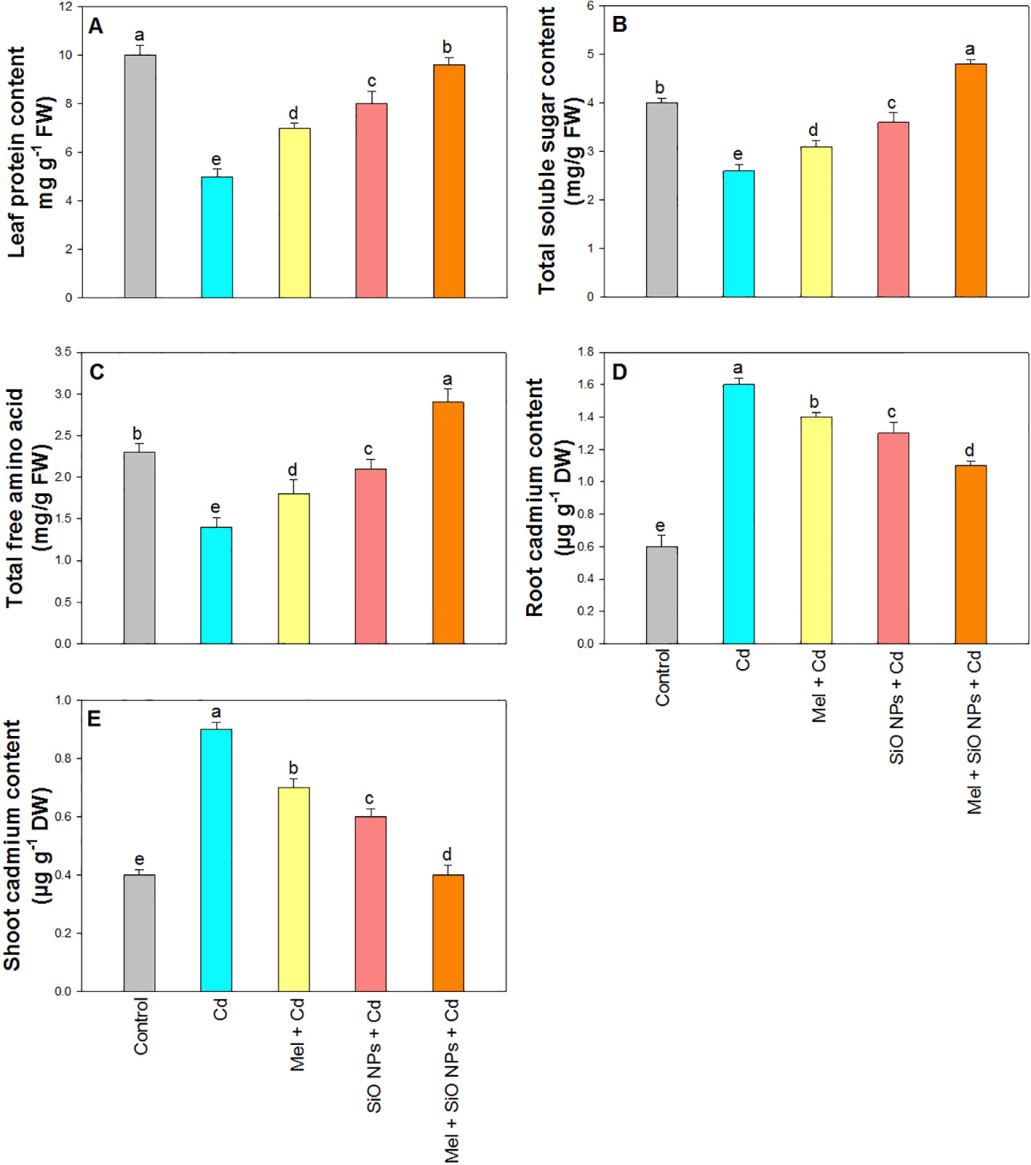
Exogenous application of SiO-NPs and Mel on 40-days old rice plants under Cd stress. **(A)** Protein content; **(B)** Total soluble sugar content; **(C)** Total free amino acid **(D)** Root Cd content; and **(E)** Shoot Cd content. The data is shown as the average of three sets ± Standard Error (S.E.). Different letters in graphs denote significant differences between control and treatment at P < 0.05 (Tukey’s test).

Protein content drastically reduced in the plants treated with Cd (**Figure 6A**). However, both SiO-NPs and Mel mitigate the toxicity of Cd stress in rice plants and enhanced protein content by 7% (SiO-NPs), 9% (Mel), and 12% (SiO-NPs and Mel) under Cd stress.

### 3.8 SiO-NPs and Mel mediated impacts on TSS and TFAA

Significant reduction was observed in TSS and TFAA under Cd stress (**Figures 6B, C**). However, the root dipping of SiO-NPs and foliar application of Mel showed an increasing tendency for TSS and TFAA. Combined treatment of both SiO-NPs and Mel produced maximum incensement in rice plants under Cd stress.

### 3.9 SiO-NPs and Mel content reduced Cd uptake to mitigated Cd stress

The significant enhancement in Cd content was observed in plants supplemented with 0.8 mM of Cd were observed in the root and shoot compared to control plants (**Figures 6D, E**). However, SiO-NPs and Mel supplementation reduced Cd uptake in root and shoot significantly. Maximum reduction was observed in the plants treated with both SiO-NPs and Mel under Cd stress (**Figures 6D, E**).

### 3.10 Stomatal response

**Figure 7:**
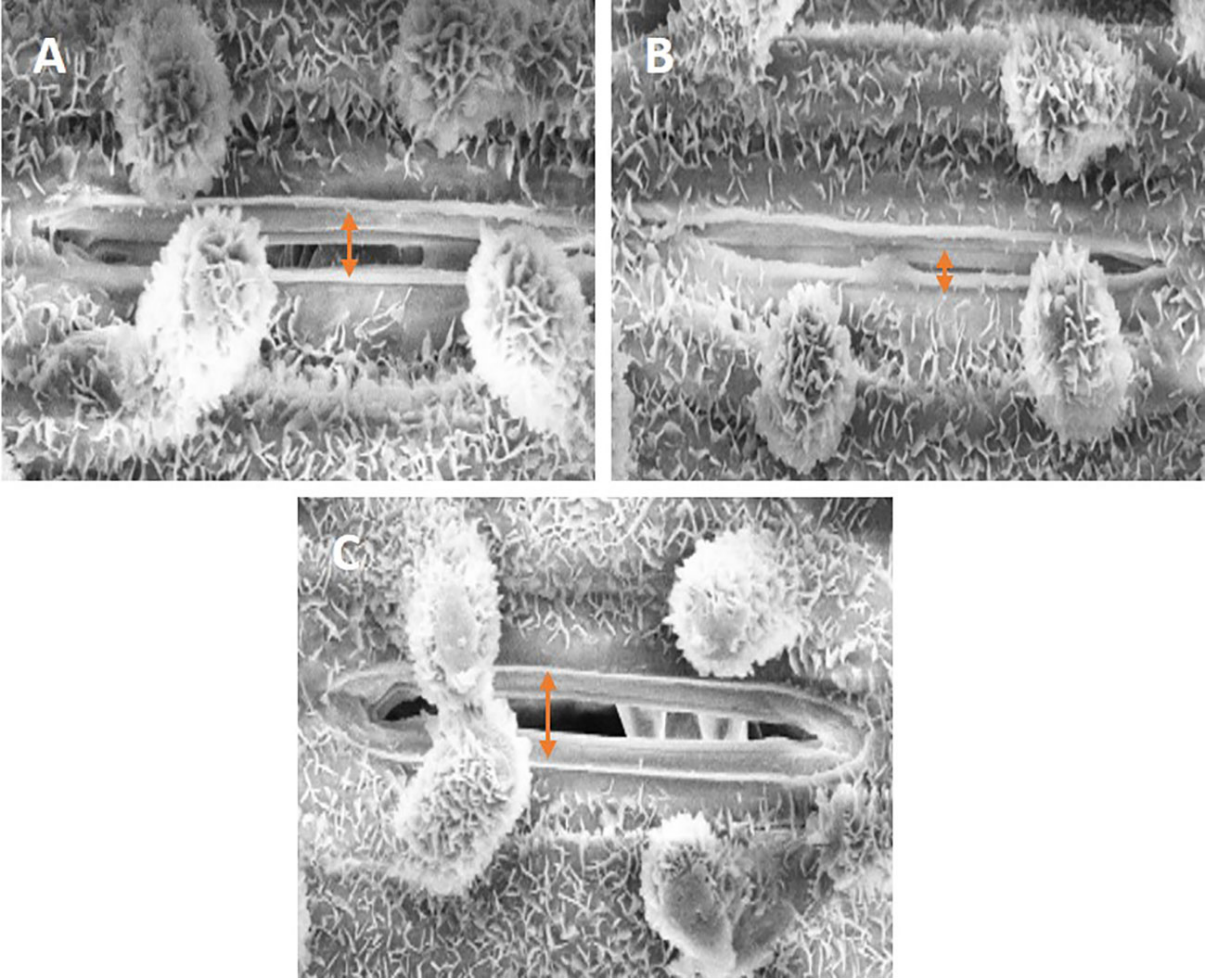
Figure represents scanning electron microscopic images of stomata of 40-day old rice plant; **(A)** control, **(B)** Cd stress **(C)** SiO-NPs (100 mg/L) and Mel (100 µm) with Cd stress.

SEM images clearly displayed a distinct difference in the stomatal aperture analysis in response to SiO-NPs and Mel when Cd was present (**Figures 7A-C**). When Cd was available to plants, the stomata’s opening decreased; however, when SiO-NPs and Mel were applied exogenously, under Cd stress, the stomatal aperture increased (**Figures 7A-C**).

## 4 Discussion

It is imperative to step up the quest for a climate-smart remediation strategy that can reduce Cd uptake and realistically create resistance to HM toxicity given the current status of the global spike in demand for agricultural production. Some intriguing results came from the studies utilizing the synergistic application of Mel and SiO-NPs to rice plants to protect them from the toxicity of Cd. Precision agriculture is undergoing a revolution through the application of nanotechnology, which will make it possible to use resources more sustainably, economically, and effectively (Hong et al., 2021). Furthermore, exogenously administered hormone-enabled strategies are also drawing attention because they appear to be promising remedies for phytotoxicity caused by HMs. Melatonin and SiO-NPs can interact directly or indirectly with plants, leading to enhanced enzymatic activity, a more robust and efficient shoot system, and higher photosynthetic efficiency as well as better crop quality (Ali et al., 2023; Huang et al., 2024). It has been demonstrated that Mel and silicon lessens the symptoms of plant toxicity caused by HMs, such as Cd (Ali et al., 2022; Wang et al., 2022). In addition, utilizing SiO-NPs is a more efficient approach to lessen Cd stress than conventional silicon-based fertilizers (Chen et al., 2018). In this investigation, the Cd-enriched rice plants experienced severe stress due to elevated Cd contents. However, SiO-NPs and Mel significantly reduced the Cd toxicity and increased the growth of rice plants (**Figures 3A-F**). Melatonin and NPs accelerated the growth rate of numerous plant species, including tomato, radish, and wheat and provide tolerance against Cd toxicity through variety of processes, such as better mineral uptake and inhibits plants’ ability to absorb Cd and lessens the harmful effects of Cd toxicity (Hussain et al., 2018; Li et al., 2016; Tang et al., 2016; Al-Huqail et al., 2020). Additional factors contributing to enhanced development and morphological characteristics could be the quantity, size, and surface area of the leaves as well as their number. Similar findings were made by Ur Rahman et al. (2020) with Si NPs and Hasan et al. (2015) with Mel, noted that under Cd toxicity, wheat and tomato seedlings produced more biomass, growth and improved root architecture. Furthermore, in Mel-pretreated watermelon seedlings exposed to vanadium toxicity, the fresh and dry weights of the root and shoot rose (Nawaz et al., 2018). In a different study, lettuce roots with graphene oxide NPs applied topically showed improved root shape (Gao et al., 2020). Melatonin inhibits HM translocation and boosts Mel gene expression, raising the endogenous concentration to combat HM stress (Faizan et al., 2024b). Applying Mel increased the growth of radish seedlings under Cd stress (Xu et al., 2020), tomato seedlings under nickel toxicity (Jahan et al., 2020), cucumber seedlings under iron toxicity (Ahammed et al., 2020), and maize seedlings under chromium toxicity (Malik et al., 2021). These findings are supported by a number of studies. Our results therefore validated the application of exogenous Mel to enhance the growth of Cd-stressed plants. According to these findings, using SiO-NPs and Mel may be a useful strategy for reducing the harmful effects of Cd exposure. It is known that SiO-NPs and Mel is novel plant growth regulators that improve several plant species’ resistance to abiotic stress.

Determining the effectiveness of plant development, particularly in stress conditions, may be aided by the efficient and rapid evaluation of photosynthetic pigments (Soni et al., 2023). The results of this study show that Mel and SiO-NPs increased the SPAD value of chlorophyll in the rice plant (**Figure 4A**). The first indication of Cd toxicity in plants is a decrease in the synthesis of photosynthetic pigments because Cd disrupts the machinery involved in photosynthetic processes, which lowers the amount of chlorophyll. The main reason for the decreased plant growth and yield might be the lower chlorophyll content. **Figure 4A** of our data showed that treated plant has higher concentrations of photosynthetic pigments over control plant. Chlorophyll concentrations rise in treated plants compared to control (**Figure 4A**), indicating that plants are growing healthily under Cd stress. This may be because plants have Mel and SiO-NPs treatment.

Photosynthesis is a physiological process that is sensitive to stress, meaning that a range of stressors can negatively affect it and its related features (Rastogi et al., 2019). The positive impacts of SiO-NPs and Mel on a variety of photosynthetic parameters, including gas exchange parameters, were directly linked to the increased growth seen under Cd stress as a result of their application (Cheng et al., 2022). This suggests that Mel and SiO-NPs could be able to mitigate the harmful effects of Cd. Furthermore, the increased chlorophyll levels (**Figure 4A**) seen in the plants treated with root dipping of SiO-NPs and foliar application of Mel further supported the increase in photosynthetic attributes (**Figures 4B-E**) that resulted from these treatments. Our study showed that rice plants under Cd stress had a considerable suppression of Ci (**Figure 4D**). These results are consistent with other studies showing that SiO-NPs root dipping and Mel foliar application together increase stomatal conductance, which rises CO_2_ generation in the cellular pore spaces. Consequently, it improves the efficiency of CO_2_ absorption and lessens the loss in photosynthetic rates caused by stress in plants with Cd by adjusting both stomatal and non-stomatal factors (Chen et al., 2023; Song et al., 2024; Faizan et al., 2021, Faizan et al., 2022; Xiaoying et al., 2022). Silicon can influence stomatal opening and shutting by improving cell wall flexibility and plasticity (Vaculík et al., 2015). The greater net photosynthetic rate seen in our study may be explained by improved gs, which promotes better CO_2_ and O_2_ exchange between the environment and the leaves of rice plants. When coupled with higher chlorophyll levels (chl a, chl b) and gas exchange machinery (P_N_, Gs, and E) under Cd stress, Si NPs have been demonstrated to increase photosynthetic efficiency in wheat plants (Akhtar and Ilyas, 2022). Si NPs also demonstrated improved gas exchange characteristics in plants growing under Cd stress, namely in *Phaseolus vulgaris* (Koleva et al., 2022) and *Triticum aestivum* L. (Ali et al., 2019). Si NPs considerably increased the leaf Pn, E, and gs of plants treated with both Cu^+^ and nano silicon in comparison to plants treated with Cu alone, according to research by Riaz et al. (2022). This may be because plants have Mel and SiO-NPs treatment. Moreover, Cd stress reduces enzyme activity and impedes effective light harvesting, which negatively impacts important photosynthetic indices including P_N_, gs, Ci, and E (Song et al., 2024).

**Figure 8:**
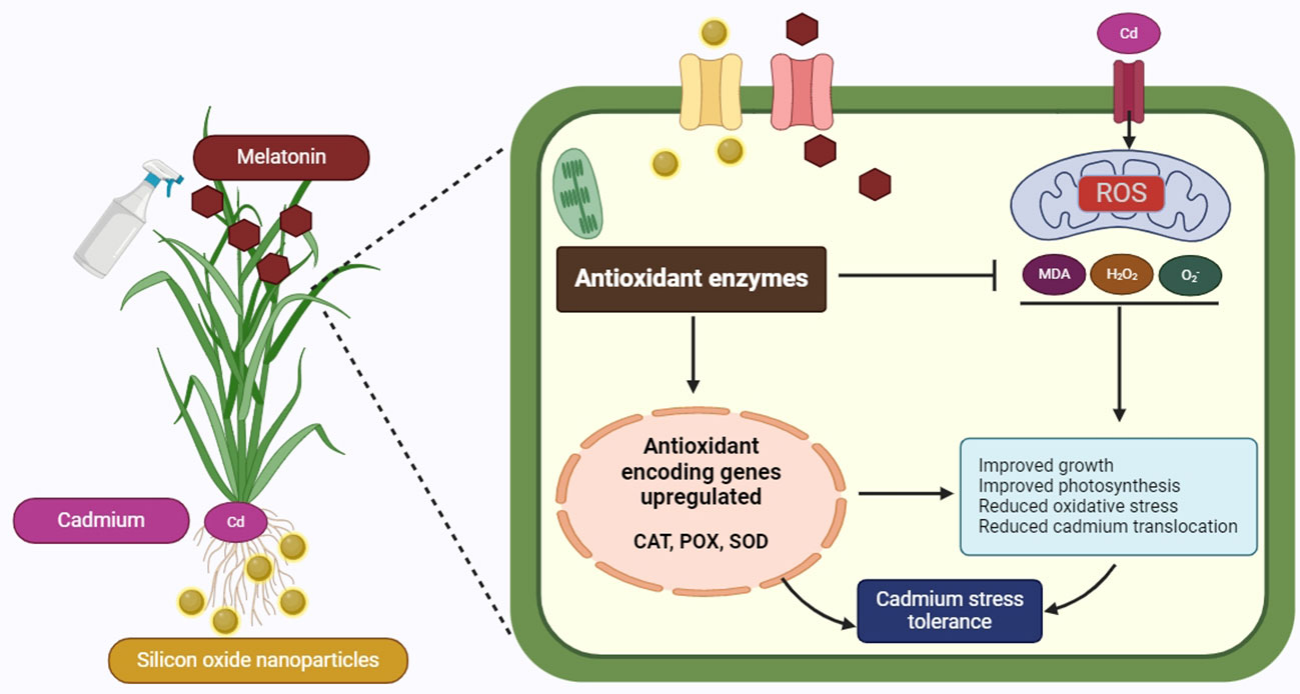
Diagram showing the possible mechanism of Cd tolerance investigated in this work using foliar spray of Mel and root dipping of SiO-NPs. [Cd, cadmium; CAT, catalase; H_2_O_2_, hydrogen peroxide; MDA, malondialdehyde; Mel, melatonin; POX, peroxidase; ROS, reactive oxygen species; SiO-NPs, Silicon oxide nanoparticles; O_2_^-^, superoxide; SOD, superoxide dismutase].

**Figure 8** presents a detailed schematic of the mechanism by which Cd tolerance is enhanced in plants through the combined use of Mel as foliar spray and SiO-NPs as root dipping. The diagram illustrates how Cd, absorbed through the roots, induces stress within the plant. Melatonin, applied to the leaves and SiO-NPs absorbed through the roots, play a crucial role in maintaining this stress. Inside the plant, the introduction of Mel and SiO-NPs leads to the upregulation of genes that encode antioxidant enzymes, specifically CAT, POX and SOD. Plants have developed complex defense mechanisms that eliminate harmful ROS from their cells using enzymes such as CAT, POX and SOD. Superoxide dismutase aids in the dismutation of O^2−^ to generate H_2_O_2_, while CAT uses a two-step process to break down H_2_O_2_ into H_2_O and O_2_ (**Figure 8**). Additionally, POX helps lipid peroxides break down (Tan et al., 2019). The activity of antioxidant enzymes was measured to confirm the generation of oxidative damage. Additionally, similar results have been found in earlier studies (He et al., 2023), which stated that HM stress was the cause of the increased ROS concentrations and the widespread generation of the H_2_O_2_ scavenging mechanism through antioxidant enzymes. In the present research, Mel and SiO-NPs could mitigate the oxidative stress toxicity caused by Cd stress, as it increased the activity of many antioxidant enzymes in rice plant under Cd stress (**Figures 5A-C**). Through the direct stimulation of antioxidative enzymes and the reduction of excessive ROS generation, Mel and NPs have the ability to shield plants from damage to their cellular membranes (Faizan et al., 2022). Silicon can alter the structure, integrity, and meanings of plasma membranes by regulating stress-induced lipid peroxidation. Silicon increased SOD activity and decreased lipid peroxidation brought on by stress, as well as stimulating root H^+^-ATPase in the membranes (Liang et al., 1999). Prior studies have demonstrated that the application of SiO-NPs enhanced the activity of antioxidant enzymes in a variety of plants, bamboo plants under lead toxicity (Emamverdian et al., 2020), rice under water regime surroundings (Elshayb et al., 2021), rice under Cd and Pb toxicity (Lai et al., 2023; Ghouri et al., 2024), wheat seedlings (Riaz et al., 2022), wheat grains at various plant development stages under Cd toxicity (Thind et al., 2021), barley seedlings under Cd toxicity (He et al., 2023). When Mel was applied to the tobacco plant under manganese toxicity, the levels of ROS were decreased, increasing the activity of antioxidant enzymes (Zhang et al., 2022). Previous research on the antioxidant system has shown an increase in response to Cd in wheat (Chen et al., 2023) and cabbage (Feng et al., 2024). It has been demonstrated that Mel and NPs increase the activity of antioxidant enzymes in *Brassica oleracea* (Anwar et al., 2024) and tomato (Song et al., 2024). Moreover, Chen et al. (2023) observed comparable outcomes, indicating that exogenous application of Mel and NPs reduced Cd stress and enhanced plant development by controlling the antioxidant system in wheat. The removal of ROS was confirmed by SiO-NPs and Mel, which resulted in a significant increase in antioxidant activity (**Figures 5A-C**). This improved activity of antioxidant enzymes leads the better growth and development of rice plant.

Proline considered as a primary metabolite in plant cells which produced under stress conditions. It shields plant from harmful conditions and speeds up their recovery from stress (Hassan et al., 2022). This osmolyte is essential for preserving photosynthetic efficiency, protecting the thylakoid membrane, regulating cellular redox potential, detoxifying ROS, stabilizing proteins and enzymes, and modifying the osmotic balance. A significant increase in proline content during Cd stress was seen in the current study, which is consistent with earlier findings (Ahmad et al., 2024). Melatonin was applied topically and SiO-NPs was dipped into the roots to further enhance proline content (**Figure 5D**). Furthermore, Mel administration increased proline accumulation and the expression of genes related to Mel formation and phytochelatin synthesis during HM stress (Sami et al., 2020; Xing et al., 2023).

The current study findings demonstrated that MDA and H_2_O_2_ levels dramatically rise in response to Cd stress (**Figures 5E, F**). The accumulation of ROS like superoxide (O^−2^), H_2_O_2_, and hydroxyl radical (OH^−^) is caused by HM stress, which also induces lipid peroxidation in terms of MDA content and oxidative stress. These ROS seriously harm the organelles, structure, and functions of plant cells (Kim et al., 2017). Silicon NPs, on the other hand, can lessen metal toxicity by decreasing lipid peroxidation, as demonstrated by a drop in MDA concentration (Alam et al., 2022). Moreover, Si NPs considerably reduce the H_2_O_2_ content when compared to the control. It has been demonstrated that Si NPs boost antioxidant enzyme activity, which reduces lipid peroxidation and mitigates the negative effects of metals like Cd (Hussain et al., 2019). In contrast, Riaz et al. (2022) demonstrated that Si NPs improved soluble proteins, polyphenol oxide, antioxidant activity, and decreased H_2_O_2_ and lipid peroxidation in plants under Cu stress. Excessive accumulation of harmful radicals such as H_2_O_2_ and O_2_ can cause lipid peroxidation, which can severely damage membrane function. But as our findings demonstrated, foliar Mel administration and root dipping with SiO-NPs decreased the production of these dangerous radicals. According to earlier research by Farooq et al. (2022) and Chen et al. (2023), these harmful radicals were decreased under stress by Mel and NPs application.

Many published research articles has demonstrated that SiO-NPs may lower the levels of Cd in a number of plants (Adrees et al., 2022; Ahmed et al., 2023a, b). Moreover, the application of SiO_2_ NPs reduces the translocation of metal from roots into shoots and decreases the metal content in rice seedlings (Chen et al., 2018). Plants that are exposed to SiO_2_ NPs have been demonstrated to absorb more Si and less Cd (Khan et al., 2020). Our results demonstrate that Mel and SiO-NPs lowers the levels of Cd in rice under Cd toxicity (**Figures 6D, E**). This exogenous application of Mel and SiO-NPs reduced the translocation of Cd to shoots; furthermore, it might be associated with increased biomass in plants under Cd stress. According to a recent study, pepper seedlings supplemented with Mel have much less accumulation of HMs (such as Cd, chromium, nickel, and vanadium) from the roots to the shoots (Altaf et al., 2023). Similar outcomes were noted for watermelon under vanadium and Cd toxicity (Saqib et al., 2023; Nawaz et al., 2018), tomato under Cd toxicity (Jahan et al., 2020), cucumber under iron toxicity (Ahammed et al., 2020), safflower under lead toxicity (Haghi et al., 2022), and wheat under Cd toxicity (Kaya et al., 2019). Cd stress reduced TSS content in rice, however exogenous application of SiO-NPs and Mel alone as well as in combination reduced the hazardous effect of Cd and increased TSS in rice plants. Our findings are consistent with prior research that found Mel plays a vital function in plant growth and development. Mel therapy increased the germination rates of *Limonium bicolor* and *Cucumis melo* L. seeds under salt stress by enhancing soluble sugar utilization, synthesis of new proteins, and increasing amylase and α-amylase activity (Li et al., 2019). More thorough investigation will be required in the future to completely comprehend this mechanism.

**Figure 9:**
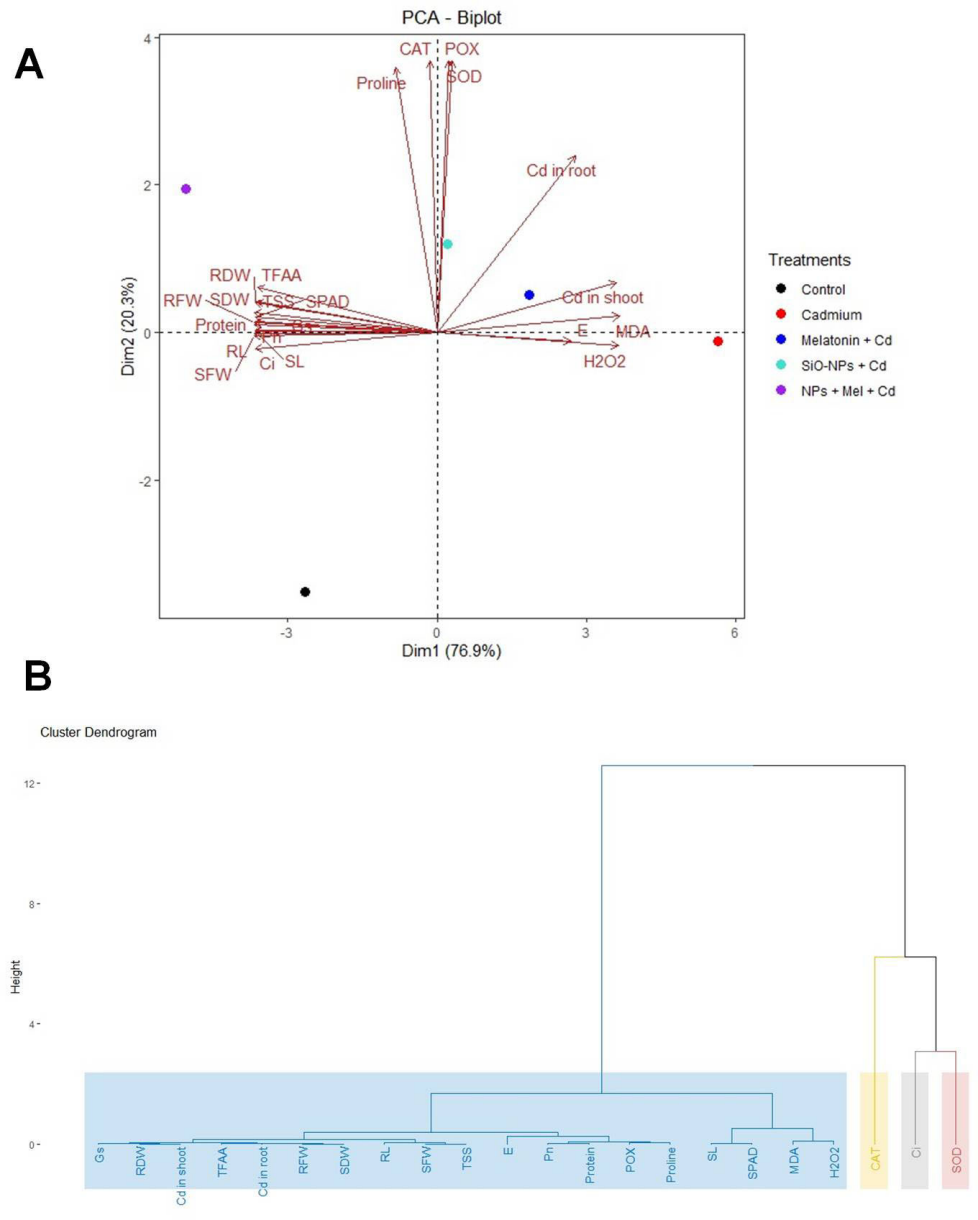
**(A)** The PCA biplot visualizes the relationships between treatments and traits based on principal component analysis (PCA). PC1 explains 76.9% of the variance, while PC2 explains 20.3%, capturing a total of 97.2% of the variability in the dataset. Each point represents a treatment, color-coded by group, and arrows indicate the contribution of traits. The length and direction of the arrows show how strongly each trait contributes to the separation between treatments, with traits like Cd in root and H_2_O_2_ driving the separation along PC1. **(B)** Hierarchical Clustering Dendrogram of traits based on their performance across all the treatments. Traits that merge at lower heights have similar patterns across treatments, indicating their similarity. The color-coded rectangles represent distinct clusters of traits, highlighting which groups of traits tend to behave similarly under different treatment conditions.

**Figure 10:**
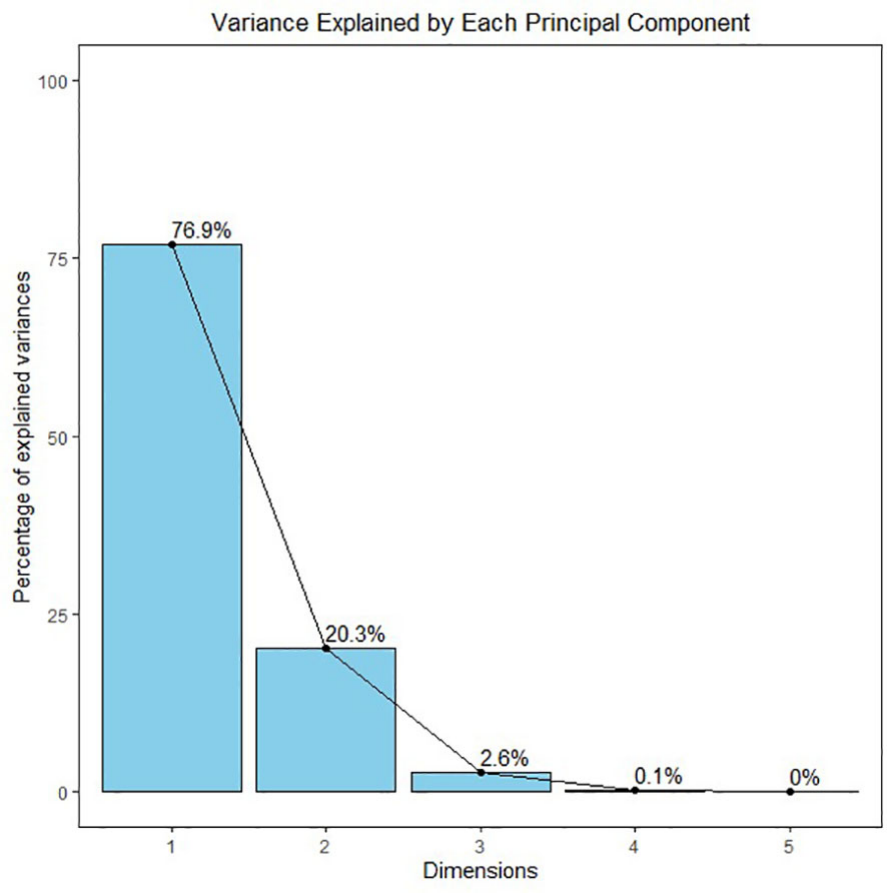
Screen plot of % variance explained by Principal Components. The plot shows the percentage of variance explained by each principal component. Sky-blue bars with black outlines represent the components, with the percentage of variance displayed on top of each bar. The first few components explain most of the variance.

**Figure 9** A showing the PCA biplot visualizes the relationships between treatments and traits based on principal component analysis (PCA). PC1 explains 76.9% of the variance, while PC2 explains 20.3%, capturing a total of 97.2% of the variability in the dataset. Each point represents a treatment, color-coded by group, and arrows indicate the contribution of traits. The length and direction of the arrows show how strongly each trait contributes to the separation between treatments, with traits like Cd in root and H_2_O_2_ driving the separation along PC1. Hierarchical Clustering Dendrogram of traits based on their performance across all the treatments. Traits that merge at lower heights have similar patterns across treatments, indicating their similarity. The color-coded rectangles represent distinct clusters of traits, highlighting which groups of traits tend to behave similarly under different treatment conditions (**Figure 9B**). **Figure 10** displaying the Screen plot of % variance explained by Principal components. The plot shows the percentage of variance explained by each principal component. Sky-blue bars with black outlines represent the components, with the percentage of variance displayed on top of each bar. The first few components explain most of the variance.

## 5 Conclusion

In summary, under Cd stress, exogenous Mel and SiO-NPs administration has been shown to enhance plant growth, biomass accumulation, and photosynthetic efficiency. As a growth-promoting substance, Mel and SiO-NPs also improved antioxidant defense system, decreased the absorption and accumulation of Cd, balanced proline, protein, TSS and TFAA level, maintained cellular integrity, and lessened oxidative damage caused by Cd. These complex effects of Mel and SiO-NPs present encouraging opportunities for its practical application in reducing the harmful effects of Cd stress on rice plants, supporting environmentally friendly farming practices. Future research and practical applications based on these findings can be used to fully investigate the potential of Mel and SiO-NPs as a helpful supplement for boosting rice resilience and ensuring the sustainability of agricultural activities in Cd-contaminated regions. Furthermore, clarifying the molecular processes underlying various stresses and crops is essential for a thorough comprehension of these protective benefits.

## Data Availability

The original contributions presented in the study are included in the article/supplementary material. Further inquiries can be directed to the corresponding author.
